# Proposed Risk Score in Patients with Aortic Stenosis Submitted to Valve Replacement Surgery

**DOI:** 10.21470/1678-9741-2022-0254

**Published:** 2023

**Authors:** Ricardo de Gasperi, Luiz Carlos Bodanese, João Carlos Vieira da Costa Guaragna, Mario Bernardes Wagner, Luciano Cabral Albuquerque

**Affiliations:** 1 Department of Interventional Cardiology, Associação Dr. Bartholomeu Tacchini, Bento Gonçalves, Rio Grande do Sul, Brazil; 2 Department of Cardiology, Hospital São Lucas, Faculdade de Medicina, Pontifícia Universidade Católica do Rio Grande do Sul, Porto Alegre, Rio Grande do Sul, Brazil; 3 Department of Cardiology, Hospital Divina, Porto Alegre, Rio Grande do Sul, Brazil; 4 Department of Postgraduate Program Stricto Sensu in Medicine and Health Sciences, Faculdade de Medicina, Pontifícia Universidade Católica do Rio Grande do Sul, Porto Alegre, Rio Grande do Sul, Brazil; 5 Department of Cardiac Surgery, Hospital São Lucas, Pontifícia Universidade Católica do Rio Grande do Sul, Porto Alegre, Rio Grande do Sul, Brazil

**Keywords:** Risk Factors, Aortic Valve Stenosis, Heart Valves, Coronary Artery Bypass, Aging

## Abstract

**Introduction:**

Due to Brazilian population aging, prevalence of aortic stenosis, and limited number of scores in literature, it is essential to develop risk scores adapted to our reality and created in the specific context of this disease.

**Methods:**

This is an observational historical cohort study with analysis of 802 aortic stenosis patients who underwent valve replacement at Hospital São Lucas, Pontifícia Universidade Católica do Rio Grande do Sul, from 1996 to 2018. With the aid of logistic regression, a weighted risk score was constructed based on the magnitude of the coeficients β of the logistic equation. Two performance statistics were obtained: area under the receiver operating characteristic curve and the chi-square (χ2) of Hosmer-Lemeshow goodness-of-fit with Pearson’s correlation coeficient between the observed events and predicted as a model calibration estimate.

**Results:**

The risk predictors that composed the score were valve replacement surgery combined with coronary artery bypass grafting, prior renal failure, New York Heart Association class III/IV heart failure, age > 70 years, and ejection fraction < 50%. The receiver operating characteristic curve area was 0.77 (95% confidence interval: 0.72-0.82); regarding the model calibration estimated between observed/predicted mortality, Hosmer-Lemeshow test χ2 = 3,70 (*P*=0.594) and Pearson’s coeficient r = 0.98 (*P*<0.001).

**Conclusion:**

We propose the creation of a simple score, adapted to the Brazilian reality, with good performance and which can be validated in other institutions.

## INTRODUCTION

Aortic stenosis presents a growing prevalence as a consequence of life expectancy increasing and natural population aging, having as a main cause the aortic calcification^[^1^,^2^]^. It is estimated a prevalence of 0.2% among adults and 2.8% in patients > 75 years of age^[^1^]^. Historically, the treatment of choice is aortic valve replacement surgery, but transcatheter aortic valve implantation (TAVI) has expanded its indications according to the latest guidelines, based mainly on the classification of surgical risk^[^2^, ^3^, ^4^]^. The importance of estimating the surgical risk in aortic stenosis patients, who are candidates for the intervention, is mandatory, because it suggests the risk of death and it also implies the kind of intervention to be performed.

The surgical risk scores most widely used and mentioned by the guidelines are the European System for Cardiac Operative Risk Evaluation (EuroSCORE) and the Society of Thoracic Surgeons (STS) Score^[^5^, ^6^, ^7^, ^8^, ^9^, ^10^]^. EuroSCORE is a death score based mostly on European patients and formed by different types of cardiac surgeries^[^8^, ^9^, ^10^]^. On the other hand, STS Score is an American morbidity and mortality score, made of three big cohorts: coronary artery bypass grafting (CABG), valve surgeries, and valve surgeries combined with CABG^[^5^, ^6^, ^7^]^. In the Brazilian reality and in the context of valve surgery, there is the Guaragna Score, a risk of death score created in a single center and already validated in other services^[^11^]^.

There are several predictors that add mortality risk to cardiac surgery, according to previous studies, such as: advanced age, female gender, diabetes, renal failure, stroke, pulmonary hypertension, advanced functional class of heart failure, endocarditis, hypertension, chronic obstructive pulmonary disease (COPD), atrial fibrillation, previous cardiac surgery, urgent and emergency surgeries, patients critical condition, and degree of ventricular dysfunction^[^5^, ^6^, ^7^, ^8^, ^9^,^11^, ^12^, ^13^, ^14^, ^15^, ^16^]^.

Due to population aging, prevalence of aortic stenosis, and limited number of scores in literature, especially in the Brazilian reality, is extremely important to develop risk scores adapted to our reality and, mainly, created in the specific scope of this pathology.

## METHODS

### Study Design

This is an observational study of a historical cohort based on the database of the postoperative cardiac surgery unit at Hospital São Lucas, Pontifícia Universidade Católica do Rio Grande do Sul (PUCRS), in accordance with the principles established in the Declaration of Helsinki and approved by the Research Ethics Committee of Faculdade de Medicina – PUCRS, under the registration number 2,796,970.

### Population

**Fig. 1:**
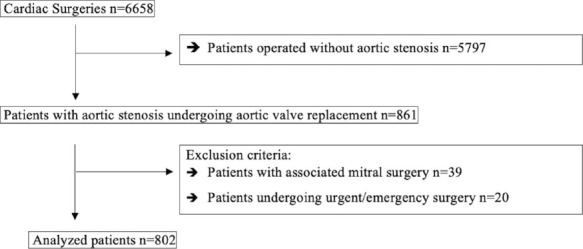
Diagram of patients included in the analysis (n=802).

Between January 1996 and July 2018, 6,658 patients underwent cardiac surgery at Hospital São Lucas, PUCRS. Of these, 802 patients aged > 18 years and with aortic stenosis who underwent aortic valve replacement alone or in combination with CABG were included in the analysis. Exclusion criteria were patients undergoing aortic valve replacement combined with another valve approach, aortic approaches or associated myectomy, and emergency/urgent surgeries (all 20 cases excluded by urgency were due to acute coronary syndrome) (Figure 1).

### Analyzed Variables

The variables initially tested in the statistical analyses were: age, gender, heart failure according to functional class by the New York Heart Association (NYHA) classification, presence of atrial fibrillation, stroke, diabetes mellitus, hypertension, previous heart surgery, chronic obstructive pulmonary disease (diagnosis through clinical data, imaging methods, spirometry, or use of continuous medications), pulmonary arterial hypertension (pulmonary artery systolic pressure > 30 mmHg), current or recent endocarditis (last 60 days), obesity (classified as body mass index ≥ 30 kg/m^2^), ejection fraction (EF) measured by echocardiography, previous chronic renal failure (creatinine ≥ 1.5 mg/dL), hemodialysis, and isolated or concomitant aortic surgery with CABG.

### Outcome

The analyzed outcome was death, being considered during the intraoperative period and throughout the hospitalization period.

### Statistical Analysis

Continuous variables were described as mean and standard deviation and compared using Student’s *t*-test, and categorical variables were described by counts and percentages, being compared by the chi-square test. The initial consideration of the variables followed a hierarchical model based on biological plausibility and previous information from the literature regarding to the relevance and strength of the associations of these potential risk factors with the occurrence of death. Once these variables were listed, multiple logistic regression was performed in a forward stepwise selection process, keeping in the model all variables with a significance level of *P*<0.05. The variable EF < 50% was also kept in the model because it is described in literature as having a strong association with the outcome. Then, a weighted risk score was constructed based on the magnitude of the β coeficients of the logistic equation. The coeficients were transformed into odds ratios (OR - exp [β]) and rounded to a whole number through the truncation process. Two performance statistics were obtained: area under the receiver operating characteristic (ROC) curve and Hosmer-Lemeshow chi-square (χ2) goodness-of-fit test with the Pearson’s correlation coeficient between the events observed and those predicted by the model as calibration estimate.

The resulting logistic model followed the formula below and, unlike the score, presents direct estimates of the probability of occurrence of the outcome.


P(events)=1/1+exp(−(β0+β1×1+…+βk×k))

Data were processed and analyzed using the IBM Corp. Released 2013, IBM SPSS Statistics for Windows, version 22.0, Armonk, NY: IBM Corp.

## RESULTS

In the study sample of 802 patients, 39.9% were female, with a mean (± standard deviation) age of 62.9 (±13.8) years and ranging from 18 to 91 years, finding a total death rate of 10.5%. The mortality of patients undergoing valve replacement alone was 5.9%, while in patients undergoing valve replacement associated with CABG (28.3% of the sample) it was 22.0%.

Table 1 shows all the variables studied with the univariate calculation analysis. After performing multiple logistic regression of variables, the following predictors obtained statistical significance for the construction of the score: aortic valve surgery combined with CABG, previous renal failure, NYHA class III/IV heart failure, and age > 70 years. The variable EF < 50% reached borderline values for statistical significance (OR 1.66, 95% confidence interval [CI] 0.96 – 2.86, *P*=0.07), and as a strong predictor associated with death in this group of patients, according to literature, it was included in the score composition (Table 2).

**Table 1:** Univariate analysis (n=802).

Variables	Deaths	Survivors	OR	95% CI	*P*-value
	n=84 (%)	n=718 (%)			
Age > 70 years	51 (60.7)	253 (35.2)	2.84	1.79-4.52	< 0.001
Female	36 (42.9)	284 (39.6)	1.15	0.73-1.81	0.560
NYHA III/IV	48 (57.1)	240 (33.4)	2.66	1.68-4.20	< 0.001
Previous cardiac surgery	0 (0.0)	8 (1.1)	-	-	0.182
Atrial fibrillation	7 (8.3)	52 (7.2)	1.16	0.51-2.65	0.722
Stroke	6 (7.1)	21 (2.9)	2.55	1.00-6.52	0.071
Diabetes mellitus	25 (29.8)	112 (15.6)	2.29	1.38-3.81	0.002
Hypertension	59 (70.2)	392 (54.6)	1.96	1.20-3.20	0.005
COPD	18 (21.4)	81 (11.3)	2.14	1.21-3.79	0.013
Pulmonary hypertension	1 (1.2)	3 (0.4)	2.87	0.29-27.92	0.409
Endocarditis	1 (1.2)	3 (0.4)	2.87	0.29-27.91	0.409
Obesity	14 (16.7)	71 (9.9)	1.82	0.98-3.40	0.073
EF < 50%	28 (33.3)	125 (17.4)	2.37	1.45-3.88	0.001
Renal failure	20 (23.8)	52 (7.2)	4.00	2.25-7.12	< 0.001
Hemodialysis	2 (2.4)	1 (0.1)	17.49	1.57-194.96	0.019
CABG associated	50 (50.9)	177 (24.7)	4.49	2.81-7.17	< 0.001

CABG=coronary artery bypass grafting; CI=confidence interval; COPD=chronic obstructive pulmonary disease; EF=ejection fraction; NYHA=New York Heart Association; OR=odds ratio

**Table 2:** Logistic regression (n=802).

Variables	Coeficient β	OR	95% CI	*P*-value
CABG associated	1.35	3.84	2.35-6.28	< 0.001
Renal failure	0.88	2.42	1.29-4.54	0.006
NYHA III/IV	0.80	2.23	1.35-3.67	0.002
Age > 70 years	0.62	1.86	1.13-3.06	0.014
EF < 50%	0.51	1.66	0.96-2.86	0.070

CABG=coronary artery bypass grafting; CI=confidence interval; EF=ejection fraction; NYHA=New York Heart Association; OR=odds ratio

At the final risk score, aortic valve surgery combined with CABG received 3 points, previous renal failure received 2 points, heart failure class III/IV received 2 points, and age > 70 years and EF < 50% received 1 point each (Table 3).

**Table 3:** Multivariate risk score (n=802).

Variables	Score
CABG associated	3
Renal failure	2
NYHA III/IV	2
Age > 70 years	1
EF < 50%	1

CABG=coronary artery bypass grafting; EF=ejection fraction; NYHA=New York Heart Association

Risk of death according to the risk score and classification (additive score) were divided into four groups — low, medium, high, and very high (Table 4).

**Table 4:** Risk of death according to the score (n=802).

Score	Sample	Deaths		Risk category
		n	%	
0	227	4	1.8	Low
1 to 3	362	24	6.6	Medium
4 and 5	127	25	19.7	High
6 to 9	86	31	36.0	Very high

**Fig. 2:**
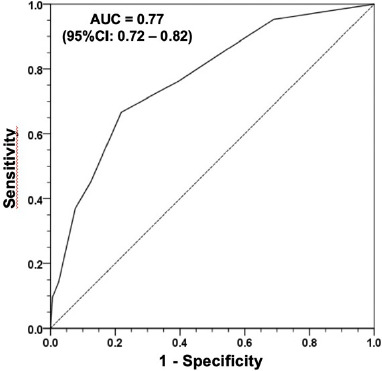
Area under the receiver operating characteristic curve in detecting the occurrence of death (n=802). Area under the curve (AUC)=0.77 (95% confidence interval [CI] 0.72- 0.82).

**Fig. 3:**
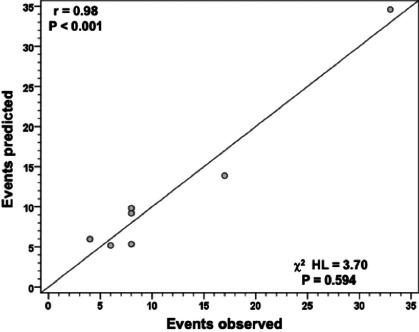
Distribution of points in the predicted and observed model (n=802). Person’s coeficient r=0.98 (P<0.001) and chi-square Hosmer-Lemeshow (χ2 HL) = 3.70 (P=0.594).

The risk model had an accuracy measured by the area under the ROC curve of 0.77 (95% CI 0.72 – 0.82) and, therefore, had good discriminatory ability. There was also a good correlation between predicted and observed mortality: r = 0.98 (*P*<0.001) with χ2 = 3.70 (*P*=0.594) (Hosmer-Lemeshow test) (Figures 2 and 3).

## DISCUSSION

This study proposed the creation of a risk score for death in patients undergoing aortic valve replacement by including five variables: aortic valve surgery combined with CABG, renal failure, NYHA class III/IV heart failure, age > 70 years, and EF < 50%.

The overall mortality obtained was 10.5%, whereas in isolated aortic valve surgery, it was 5.9%. Compared to literature data, especially in relation to European and North American data, it was reported a high overall mortality rate. When it was compared with STS Score cohort that involves only isolated valve surgery, it was observed a mortality of 3.4%, and 5.6% in the cohort associating aortic valve surgery with CABG^[^5^,^6^]^. New York State cohort describes a 3.3% mortality for isolated aortic valve replacement and 7.1% for aortic valve replacement associated with CABG^[^15^]^. The United Kingdom data from the Ambler Score cohort shows a mortality rate of 4.9% for aortic valve replacement and 7.9% for combined surgery with CABG^[^14^]^. When national cohorts are analyzed, some numbers closer to this study’s results can be seen. Garofallo et al.^[^17^]^ presented data from a tertiary center in the same city, also involving patients’ interventions from the public and private healthcare system, and reported 8.6% mortality for valve surgery and 20% when associated with CABG. Ribeiro et al.^[^18^]^, reviewing data from > 100,000 surgeries performed in Brazil between 2000-2003, described a mortality of 8.9% for valve surgeries and 16.5% for associated surgeries. Bueno et al.^[^19^]^ presented data from the 1990s showing mortality for isolated aortic surgery of 8% and 21% for aortic surgery associated with CABG. The Brazilian registrY of adult Patients undergoing cArdiovaScular Surgery (or BYPASS), a recent Brazilian registry, organized by the Brazilian Society of Cardiovascular Surgery analyzing data on valve surgeries from 920 patients (80% from the public and 20% from the private healthcare system of 17 different institutions in the country), found a mortality in aortic valve replacement isolated of 5.1% and 14.7% in aortic valve replacement associated with CABG. In this analysis, when only interventions in aortic degenerative disease were evaluated, the mortality was 7.8%^[^20^]^. It is worth emphasizing that this analysis included data from more than twenty years of interventions, representing nearly three decades and covering different stages of cardiac surgery within PUCRS hospital. Analyzing the same period, another national reference center in cardiology reported mortality in valve surgery ranging from 7.47% to 13.96%^[^21^]^.

According to the studies, it was observed that in aortic valve replacement surgery combined with CABG compared to isolated valve replacement, there is an increased risk of death. Based on the abovementioned national studies, an increase of at least two to three times in the risk of death for combined surgery can be seen^[^17^,^18^,^20^]^. Regarding to STS Score and the United Kingdom cohorts, it was observed a less than twofold increase in associated surgery mortality compared to isolated aortic valve, and in New York State cohort, it was twice as high^[^5^,^6^,^14^]^. It was found in this study that combined surgery was responsible for adding 3 points (OR 3.84) to the risk of death, being the main score risk predictor. Patients with only this variable would receive 3 points, being moderate risk with an estimated mortality of 6.6%. Since mortality in valve surgery associated with CABG in study cohort was much higher, most patients undergoing this intervention presented an association of risk variables. It is highlighted that most of the national services, as well as our service, mostly assisted patients are from the public healthcare system — patients who often have serious socioeconomic problems, difficulties in optimal use of drug treatment, less access to medical care, as well as being in more advanced stages of the disease when undergoing surgical intervention^[^17^]^.

Renal failure is another common risk factor across all different risk scores^[^5^, ^6^, ^7^, ^8^, ^9^,^11^, ^12^, ^13^, ^14^, ^15^, ^16^]^. In this study, it was defined as a creatinine ≥ 1.5 mg/dL and it was responsible for adding 2 points to the mortality risk. There is a wide variation in the definition of renal failure within heart surgery cohorts. It is known that small increases in creatinine above normal levels and a slight decrease in the glomerular fltration rate are already associated with a worse prognosis in cardiac surgery^[^22^,^23^]^. In a previous study published by this hospital group, two different risk levels were aggregated — the first, when creatinine was between 1.5 and 2.49 mg/dL, and a second, with a higher risk level, when creatinine was ≥ 2.5 mg/dl or on chronic dialysis^[^11^]^. In the cohort study, there were only three patients on chronic dialysis, compromising the analysis of this variable as being an isolated risk predictor.

In a review by Tjang et al.^[^24^]^, NYHA class III/IV heart failure was the most mentioned risk factor among the predictors of mortality in patients undergoing aortic valve replacement. Functional class in heart failure is a strictly clinical factor, with simple assessment and easy applicability, supporting the idea that patients with aortic stenosis should undergo valve replacement before their clinical deterioration. In the study score, the advanced functional class III/IV added 2 points to the increased risk of in-hospital death, and 36% of patients were in this class. Regarding to the STS Score cohorts, there was a division of risks into two groups: first, a lower risk group involving functional classes I to III, and a second higher risk group including only functional class IV^[^5^,^6^]^. The Ambler Score^[^14^]^ and EuroSCORE^[^10^]^ do not include the functional class as a risk predictor, only the degree of ventricular dysfunction. In EuroSCORE II, the functional class was incorporated, being divided into II, III, and IV according to NYHA^[^9^]^.

Age is a risk predictor mentioned by all risk scores in literature, and the cutof point, which increases the risk of surgical mortality, varies among them^[^5^, ^6^, ^7^, ^8^, ^9^,^11^, ^12^, ^13^, ^14^, ^15^, ^16^]^. EuroSCORE determines an increased risk for mortality in patients > 60 years of age and adds an increase in risk every five years above this cutof point^[^8^]^. STS Score^[^5^, ^6^, ^7^]^ determines an increase in risk over the age of 50, and the New York State cohort^[^15^]^ shows an increase in risk every decade over the age of 55 years. Although the surgical risk increases with advanced age compared to younger patients, the benefit in relation to medical treatment is substantially advantageous. Octogenarian surgery candidates who, for their own reasons, choose not to undergo the intervention have a mortality increase greater than ten times compared to patients who are intervened. This shows that advanced age by itself is not a contraindication to valve replacement surgery^[^25^]^. It is also true that less invasive approaches such as TAVI and hybrid procedures with stents associated with TAVI must be considered within a general context, including age as well. The latest American Guideline (American Heart Association/American College of Cardiology) on valvulopathy places TAVI superior over valve replacement surgery in patients over 80 years of age, regardless of surgical risk^[^4^]^, and The Brazilian Guideline on Valvular Heart Disease ^[^2^]^ considers TAVI implantation as a class IA indication in patients over 70 years of age, even at low surgical risk.

Ventricular dysfunction is a marker of severity in the valve surgery. EuroSCORE established two risk groups for the outcome death in cardiac surgery according to EF — EF values between 30 and 50% with an OR of 1.5 and EF < 30% with an OR of 2.5^[^10^]^. In EuroSCORE II, the division of EF into more categories was prioritized, with EF 20-29% and < 20% being added^[^9^]^. STS Score in the cohort of valve surgery establishes an OR of 1.09 for each decrease of 10 EF units below 50, regardless of the kind of valve surgery^[^5^]^. Ambler score showed an OR for death of 1.2 for EF of 30-50% and an OR of 1.99 for EF < 30% when compared to EF > 50%^[^14^]^. Guaragna et al.^[^11^]^ found an OR of 2.1 for the variable EF ≤ 45 in patients undergoing valve surgery. Specifically in patients with aortic stenosis, the German Aortic Valve Score established an OR for mortality of 1.96 in patients with EF between 30 and 50% and OR of 2.96 for EF < 30%^[^12^]^. In this study, in the multivariate analysis, the variable EF < 50% reached borderline values in relation to its statistical significance (OR 1.66, 95% CI 0.96 – 2.86, *P*=0.07) and, due to its strong association described in literature, it was included in the final score, receiving 1 point. Only twenty-four patients underwent a replacement valve surgery with EF < 30%.

The surgical risk was divided into four groups, ranging from low to very high. It was emphasized that, in this sample, any of these predictors alone places the patient at medium risk and adds an important increase in the risk of death compared to the low-risk group where none of these predictors is present.

The model had an accuracy measured by the area under the ROC curve of 0.77 (95% CI 0.72 – 0.82) and, therefore, had a good discriminatory ability. There was also a good correlation between predicted and observed mortality — r = 0.98 (*P*<0.001) with χ2 = 3.70 (*P*=0.594) (Hosmer–Lemeshow test). Table 5 summarizes the main scores in literature, showing that this risk score, created from a specific group of patients with aortic stenosis, presents a superimposable accuracy.

**Table 5:** Scores’ accuracy comparative.

Score	AUC ROC
Gasperi	0.77
Guaragna Score^[^11^]^	0.83
Kotting (German Aortic Valve Score I)^[^12^]^	0.80
Nashef (EuroSCORE II)^[^9^]^	0.80
Mejía (Inscor)^[^16^]^	0.79
Ambler^[^14^]^	0.77
Roques (EuroSCORE)^[^8^]^	0.76
O’Brien (STS Score – AVR valve)^[^5^]^	0.76
Hannan^[^15^]^	0.76
Shahian (STS Score – AVR + CABG)^[^6^]^	0.74
Schiller (German Aortic Valve Score II)^[^13^]^	0.74

AUC=area under the curve; AVR=aortic valve replacement; CABG=coronary artery bypass grafting; EuroSCORE=European System for Cardiac Operative Risk Evaluation; ROC=receiver operating characteristic; STS=Society of Thoracic Surgeons

### Limitations

Related to the study limitations. Firstly, patients in need of urgent surgery were excluded from the analysis. The proposal was to create a score focused on the pathology of aortic stenosis, and the twenty cases of urgent surgery were performed on an emergency basis due to acute coronary syndrome. Secondly, variables such as previous hemodialysis, current or recent endocarditis, pulmonary hypertension, and previous cardiac surgery included a very low absolute number of patients in the sample and limited a better analysis of these variables as risk predictors. Thirdly, the risk model was created by analyzing data obtained from a single center without an internal validation group. According to literature, they tend to show lower results when applied to institutions other than where the scores were created. Thus, it is understood that this study has internal validity, however, the study researchers consider it is important a future external validation of this model in other institutions.

Regarding to practical implications of these findings, the identification of risk factors and the creation of a death risk score allow to accurately estimate the surgical risk within the institution, monitor the care quality, and implement measures for service qualification. The model has a good statistical performance and, therefore, has adequate capacity to be tested and validated in other institutions.

## CONCLUSION

In this study, it is proposed an in-hospital death risk score for patients undergoing aortic valve replacement using five variables: valve replacement surgery combined with CABG, previous renal failure, presence of NYHA class III/IV heart failure, age > 70 years, and EF < 50%. Also, this is a simple score, with good statistical performance, and adapted to the Brazilian reality.
